# Green synthesis of copper oxide nanoparticles using *Ephedra Alata* plant extract and a study of their antifungal, antibacterial activity and photocatalytic performance under sunlight

**DOI:** 10.1016/j.heliyon.2023.e13484

**Published:** 2023-02-04

**Authors:** Afrah Atri, Mosaab Echabaane, Amel Bouzidi, Imen Harabi, Bernabe Mari Soucase, Rafik Ben Chaâbane

**Affiliations:** a Laboratory of Advanced Materials and Interfaces (LIMA), Faculty of Sciences of Monastir, University of Monastir, Avenue of the Environment, 5000 Monastir, Tunisia; b NANOMISENE Lab, LR16CRMN01, Centre for Research on Microelectronics and Nanotechnology CRMN of Technopark of Sousse, B.P. 334, Sahloul, 4034 Sousse, Tunisia; c University Yahia Fares of Medea Urban Pole, Laboratory of Biomaterials and Transport Phenomena (LBMPT), (26000), Medea, Algeria; d School of Design Engineering, Universitat Politecnica de Valencia, Cami de Vera, Spain

**Keywords:** CuO-NPs, Biosynthesis, *Ephedra Alata*, Photocatalytic, Antibacterial, Antifungal activity

## Abstract

In the present work, copper oxide (CuO NPs) was synthesized by an eco-friendly, simple, low-cost, and economical synthesis method using *Ephedra Alata* aqueous plant extract as a reducing and capping agent. The biosynthesized CuO-NPs were compared with chemically obtained CuO-NPs to investigate the effect of the preparation method on the structural, optical, morphological, antibacterial, antifungal, and photocatalytic properties under solar irradiation. The CuO NPs were characterized using X-ray diffraction (XRD), UV-VIS spectroscopy, Fourier transform infrared spectrometer (FTIR) analysis, and field emission scanning electron microscopy with energy dispersive X-ray spectroscopy (FESEM-EDX). The photocatalytic activities of biosynthetic CuO-NPs and chemically prepared CuO-NPs were studied using methylene blue upon exposure to solar irradiation. The results showed that the biosynthesized CuO photocatalyst was more efficient than the chemically synthesized CuO-NPs for Methylene Blue (MB) degradation under solar irradiation, with MB degradation rates of 93.4% and 80.2%, respectively. In addition, antibacterial and antifungal activities were evaluated. The disk diffusion technique was used to test the biosynthesized CuO-NPs against gram-negative bacteria, *Staphylococcus aureus* and *Bacillus subtilis*, as well as *C. Albicans* and *S. cerevisiae*. The biosynthesized CuO-NPs showed efficient antibacterial and antifungal activity. The obtained results revealed that the biosynthesized CuO-NPs can play a vital role in the destruction of pathogenic bacteria, the degradation of dyes, and the activity of antifungal agents in the bioremediation of industrial and domestic waste.

## 1 Introduction

In recent years, in the field of nanotechnology, metal and metal oxide nanoparticles have been the most developed materials due to their modified and adjustable morphology, physicochemical properties, small size, and enormous surface area [1,2]. They find various applications such as agricultural research, pharmaceuticals, biological, and environmental. Among the various metal and metal oxide nanoparticles, copper oxide (CuO) nanoparticles have a wide and direct band gap of 1.2–2.1 eV, are a member of group II-VI of p-type semiconductors and monoclinic structures. They have become a potential candidate for environmental applications [3,4]. Additionally, they exhibit low toxicity, high chemical and thermal stability, compatibility, easy synthesis route, variable morphology at the nanoscale, a high specific surface area, improved oxygen adsorption capacity, and low cost [[5], [6], [7]]. In recent years, many well-defined CuO nanostructures have been synthesized, such as nanospheres [8], nanoflowers [9], micromachines [10], nano leaves [11], nanorods [12], nanotubes [13], nanosheets [14], and nanorings [15] to improve the surface reactivity and properties of nanoparticles. CuO-NPs are synthesized by various chemical and physical methods. But these techniques are high-cost, require long-term growth, and multi-step procedures, and are not environmentally friendly. Hence, researchers are attempting to develop clean, non-toxic and cost-effective methods that totally reduce the use of hazardous chemicals [[16], [17], [18]]. The biosynthesis methods of CuO-NPs have been indicated by various plants such as *Punica granatum peel* [19], *Cedrus deodara* [20], *Ailanthus altissima leaf* [21], *Bougainvillea flower* [22], *Cucumis sativus* (cucumber) [23] and *Brassica oleracea* var. *italic* [24]. The *E. alata* plant belongs to the family of Euphorbiaceae, and it is mostly found in China and most Arab countries. This plant has been used traditionally as a medicine to treat allergies, nasal congestion, flu, asthma, colds, chills, fever, coughs, headaches, and edema. Furthermore, the local community has a long history of using the plant's dried stems for medicinal purposes, particularly for its anti-cancer, anti-asthmatic, and bronchodilator properties. In Tunisia, *E. alata* (alenda) is a perennial, stiff shrub that grows to a height of 5 m. It is light green, densely branching, and lacks leaves. It is spread throughout southern Tunisia and is thought to be among the most severely xerophytic species. Phytochemical screening of the plant shows that a wide range of E. alata natural products, including alkaloids, tannins, saponins, proanthocyanidins, phenolic acids, flavonoids, polyphenols, and essential oils, are derived from the plant [25].

In this regard, green synthesis procedures have recently been carried out to produce NPs from biological sources because plant extracts play the role of reservoirs of phytochemicals such as polyphenols, flavonoids, proteins, vitamins, and alkaloids, which act as recovery, reducing, stabilizing, and capping agents [26,27]. CuO-NPs have a wide range of applications, including sensors [28], electrochemical [29], solar cells [30], optoelectronics [31], medical and pharmacy [32], industrial [33], and agriculture [34]. They have also received growing interest in the fields of degradation and removal of the dyes and biological activity from wastewater to reduce their impact on the environment [35]. Dye plays a vital role in many textile industries, such as food production, agricultural research, pharmaceuticals, paper production, cosmetics, and leather [1,36]. Among the chemically synthesized dyes, cationic dyes (MB) are the most commonly produced in larger quantities [37]. They can cause serious risks to human health and the natural environment due to their low biodegradability, stability, toxicity, and hazardous potential [[38], [39], [40], [41]]. For water treatment, several methods like adsorption, reverse osmosis, foculation, coagulation, evaporation, electro-precipitation, and photocatalytic degradation use UV and solar irradiation [[42], [43], [44]]. From these methods, photocatalysis has proven to be an important technique for wastewater treatment. In most cases, the development of the photocatalytic technique focuses on irradiation with sunlight compared to artificial UV radiation, due to the cost-effectiveness of the process by using a renewable solar energy source [45,46]. Moreover, the interest is to use sunlight, which is an effective, very economical, free, easy, and inexhaustible method to remove dyes, because solar energy is naturally available [47,48]. CuO is one of the most important catalysts employed for removal of industrial effluents from the environnement. The oxygen surface lattice of CuO is thought to be involved in the catalytic reaction, which appears to be a structure-sensitive mechanism. The catalytic reactivity of CuO nanostructures is influenced by their form and exposed crystal planes. Therefore, the structurally controlled synthesis of CuO structures may help develop novel structures with the needed improved performance [49,50]. Many researchers have been interested in using copper oxide nanoparticles as agents with antimicrobial activity against Gram-positive and Gram-negative bacteria as well as anti-fungal activity. In a previous study, Selvam et al. found that copper oxide nanoparticles in *Canthium coromandelicum* leaves degraded MB by 91% in 180 min [51]. The biosynthesized CuO-NPs using *Calotropis procera* leaf extract have recently attracted antibacterial and antifungal activity [52]. Karuppannan, S.K. et al. have reported that the *Cardiospermum halicacabum* aqueous extract-mediated synthesized CuO-NPs were examined under Gram-positive bacteria and MB dye was degraded by 93% in about 210 min [50]. In this work, we report on a simple, cost-effective, and green synthesis of CuO-NPs using the plant extract *Ephedra alata* for environmental, biological, and agricultural applications. Then, using X-ray diffraction (XRD), Fourier transform infrared spectroscopy (FTIR), and field emission scanning electron microscopy with energy dispersive x-ray spectroscopy (FESEM-EDX) analysis, the structural properties of the *E. alata* plant extract were studied. This extract is enriched in phenolic and flavonoid groups, which act as reducing, stabilizing, and capping agents. In addition, we discussed the photocatalytic degradation parameters of the methylene blue (MB) dye of CuO-NPs and tested the activity against *S. aureus* and *B. subtilis*, as well as *C. Albicans* and *S. cerevisiae* as bacterial and fungal spots. The novel biosynthesized CuO-NPs may be a promising candidate for environmental and biomedical applications.

## 2 Materials and methodology

### 2.1 Materials

*E. alata* plants (local name: alenda), identified by Prof. Mohamed Neffati of the Institute of Arid Lands Mednine-Tunisia, were collected in Mednine, south of Tunisia in summer. Copper sulfate pentahydrate (CuSO_4_, 5H_2_O) and cationic dye (MB) were used. The chemical structure and characteristics of the dye are shown in Fig. S1 and Table S1. Deionized (DI) water and double-distilled water (DDW) were used throughout the experiment. All the chemicals were purchased from Sigma-Aldrich without further purification.

### 2.2 Green synthesis of CuO-NPs

**Fig. 1:**
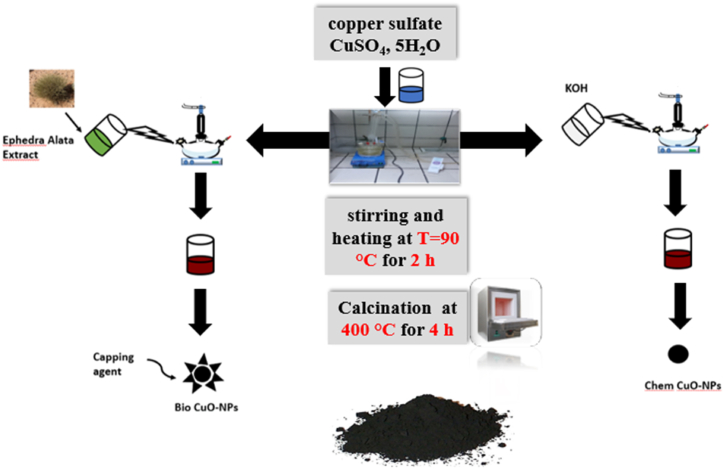
Schematic for a step-wise synthesis procedure of CuO NPs by two different routes green and chemical. (For interpretation of the references to color in this figure legend, the reader is referred to the Web version of this article.)

The dried aerial parts were crushed using an electric mill. The extraction was prepared by the infusion method. *E. alata* powder was weighed at 45 g and put into Whatman filter papers, and then 120 ml of distilled water was added. The extraction was carried out at ambient temperature for a week, and the color of the aqueous solution changed from watery to brick red. For the synthesis of CuO nanoparticles, 30 ml of *E. alata* extract was taken and boiled at 90 °C 1 g of copper oxide pentahydrate (CuSO_4_.5H_2_O) was dissolved in 10 ml of a deionized water mixture and kept under agitation for 15 min at room temperature. When the temperature of the extract reached 90 °C, the copper oxide solution was added. The resulting mixture was stirred with a magnetic stirrer for 2 h at T = 90 °C. The paste was centrifuged at 3500 rpm for 10 min, three times, and then washed with doubly distilled water. The final product was dried in an oven at 40 °C for 24 h. Finally, the CuO nanoparticles were calcined at 400 °C for 4 h (Fig. 1).

### 2.3 Chemically synthesis of CuO-NPs

The hydrothermal method was used to synthesize CuO-NPs. First, 1 g of copper sulfate, CuSO_4_, 5H_2_O, was dissolved in 40 ml of deionized water (DIW) and stirred for a few minutes on the magnetic stirrer to obtain a homogeneous, blue-colored solution. After that, 1 g of KOH was dissolved in 90 ml of DIW and the potassium hydroxide was added drop by drop to the solution until the pH reached 10. When the potassium hydroxide is injected, the color of the solution starts to change. The mixture was stirred for 2 h at T = 90 °C. The blue solution changes to a dark-colored precipitate. In addition, the precipitate obtained was centrifuged at 3500 rpm for 10 min, three times, and then washed with doubly distilled water (DDW). The black product was dried at 80 °C for 18 h. Finally, the powder was calcined at 400 °C for 4 h (Fig. 1).

### 2.4 Photocatalytic activity

The solar photocatalytic activity of these synthesized CuO NPs was evaluated by the degradation of MB. First, 25 ml of methylene blue solution (10 mg/L) was added with 20 mg of CuO NPs. The mixture was stirred magnetically for 2 h in darkness to reach the adsorption-desorption equilibrium. Afterwards, the suspension was exposed to direct sunlight for 180 min (3 h). The sunlight degradation of the dye was determined by measuring the reduction in the intensity of MB at *λ*_max_ = 666 nm using a UV-VIS spectrometer at different time intervals. The degradation of dye percentage (R) was determined using Eq. (1):

(1) $$R=((A_{0}–\;A_{t}\;/\;A_{0})\times 100$$

where A_0_ is the initial absorbance of a dye and A_t_ is the absorbance at time t.

### 2.5 Antibacterial and anti-fungal activity

The in vitro antibacterial activity of the synthesized compounds was investigated by using the disc diffusion technique. Bacteria samples from one over-night grown colony were suspended in a test tube containing physiological water. The turbidity (expressed as optical density OD) of the microorganism suspensions was measured with an optical spectrophotometer (wavelength = 625 nm) and adjusted to 0.6 to 0.8. A sterilized cotton swab was immersed in the resulting suspension, and a lawn of bacteria was applied onto the surface of the plates containing Tryptic Soy Agar for bacteria and Sabouraud dextrose agar for the fungal sample. Then, sterile filter paper disks (diameter 6 mm, Whatman paper n^o^3) were first impregnated with 30 μl of each dimethylsulfoxide (DMSO) extract (6 mg/ml). The side containing the particles was facing downwards to ensure direct interaction with the agar and microorganisms. The bacterial plates were incubated at 37 °C for 24 h, and the fungal plates were incubated at 25 °C for 48 h. Finally, the diameter of the growth inhibition zones was measured using a zone inhibition reader (Fisher Lilly Antibiotic Zone Reader). Disks with 30 μl of distilled water were used as negative controls, and antibiotic discs were used as positive controls such as Nystatine (10 mg/ml), Gectapen (40 mg/ml), Azimycine (30 mg/ml). Each experiment was carried out twice.

### 2.6 Characterisation of nanoparticles

The crystalline size and crystal structure of the CuO Nps was established by X-ray diffractometer using monochromatic Cu-Kα radiation of wavelength λ = 1542 Å from an angle 2θ = 30°–80° XRD measurements were performed using a Philips X′ Pert PRO MPD diffractometer equipped with a CuKα radiation source (λ = 1542 Å). Morphology and size distribution of the CuO Nps were observed by field emission scanning electron microscopy (SEM). Optical absorption spectra of CuO Nps were studied in a quartz cuvette by UV–Vis SPECORD 210 plus spectrophometer in the wavelength range of λ = 200–800 nm. The extract and CuO NPs were analyzed by FTIR from 4000 to 400 cm^−1^. FTIR spectra were recorded using a PerkinElmer version 5.3 spectrophotometer at room temperature in the spectral range of 400–4000 cm− 1 using KBr pellet disks. Field Emission Scanning Electron Microscopy (FE-SEM) The elemental composition of the samples was determined using energy dispersive X-ray spectroscopy (SEM-EDX), the EDX detector is a Zeiss ULTRA 55 model equipped with an In-Lens SE detector.

## 3 Results and discussion

### 3.1 X-ray diffraction analysis

**Fig. 2:**
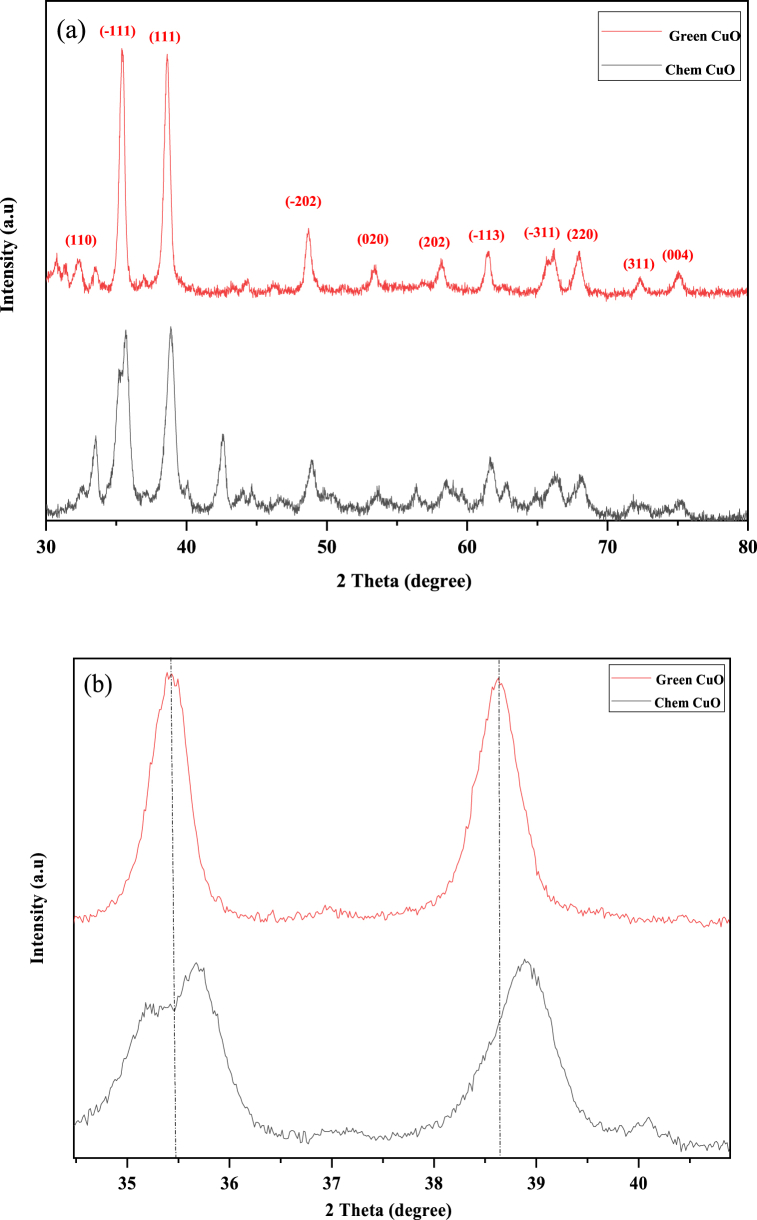
a) X-ray diffraction patterns of chem CuO-NPs and biosynthsized CuO-NPs.b). the shift of peak position of chem CuO-NP and biosynthesized CuO-NP.

The crystal structure, crystallinity, and size of the crystallite of the nanoparticles were determined by X-ray diffraction. The XRD diagrams of chemical CuO-NP and biosynthetized CuO-NP are shown in Fig. 2a. The diffraction peaks observed over 2θ of 32.53, 35.55, 38.75, 48.70, 53.54, 58.33, 61.55, 66.22, 68.15, 72.42, and 75.04° correspond to Miller indices (110) (−111) (111) (−202) (020) (202) (−113) (−311) (220) (311) (004) respectively. The observed peaks confirm the monoclinic structure of CuO-NPs, which corresponds to JCPDS map n° 89–5899. Similar results were obtained for CuO-NPs synthesized from an extract of *P. Pyrifolia* leaves and an extract of *Urtica dioica* leaves [53,54]. Additionally, no impurity peaks were observed in the diffractogram of the biosynthesized CuO-NPs, indicating the phase purity. However, for the chemical CuO-NPs, it is seen as a small peak was seen at 42.5° in the spectrum. This can be attributed to the presence of impurities, which is characteristic of Cu_2_O [55, 56]. Moreover, the diffraction peaks of the biosynthesized CuO-NPs were sharp and well defined with high intensities, which indicates the good crystallinity. However, the corresponding chemical CuO-NPs peaks present lower intensities.

The two most intense diffraction peaks (−111) and (111) of the biosynthesized CuO-NP showed a positional shift to the left with respect to the chemical CuO-NP (Fig. 2b). This shift is due to the function of phytochemicals present in the extract of *E. alata* plant [17]. The average sample size was determined by Debye Scherer's Eq. (2) [57]:

(2) $$D=\frac{k*\lambda }{\beta \;\cos\;\theta }$$

where D is the crystallite size (Å), λ is the wavelength of the incident X-rays equal to 1.54 Å, k is a constant of 0.9, β is the full width at half maximum (FWHM) and θ is Bragg's angle. The average size of the chem CuO-NP and biosynthesized CuO-NP grains was found to be 9.23 and 15.21 nm, respectively. From Table 1, it was observed that the size of biosynthesized CuO-NP increased relative to the chemical CuO-NP. This may be due to the effect of the extract affecting the parameters and volume of the lattice, resulting in the increase of the grain [18]. The microdeformation ε and dislocation density δ of chemical CuO-NPs and biosynthesized CuO-NPs were determined using the following Eq. (3,4) as [6]:

(3) $$\delta =\frac{1}{D^{2}}$$

(4) $$\varepsilon =\frac{\beta \;\cos\;\theta }{4}$$

**Table 1:** Compared of optical and structural parameter of chemical CuO-NP and biosynthesized CuO-NP.

samples	Bandgap energy (ev)	Dsh (nm)	Microstrain (*ε*)*10 ${}^{-3}$	dislocation density (δ) * $10^{15}$
Chem CuO-NP	1.92	9.23	1.7	11.7
Bio CuO-NP	1.77	15.21	1.03	4.32

The values of microstrain and dislocation density are shown in Table 1. As can be seen, the microdeformation decreased with the increasing size of the CuO-NPs, and the dislocation density is inversely proportional to the size. The dislocation of biosynthetized CuO-NPs was lower than that of the chemical CuO-NPs. This indicates that biosynthetized CuO-NPs have a higher crystallization [17]. It can be concluded that the extract plays a very important role in reducing the microdeformation and dislocation density. This proves that the biosynthetized CuO-NPs can be used as promising candidates for photocatalysis applications.

### 3.2 FTIR analysis

**Fig. 3:**
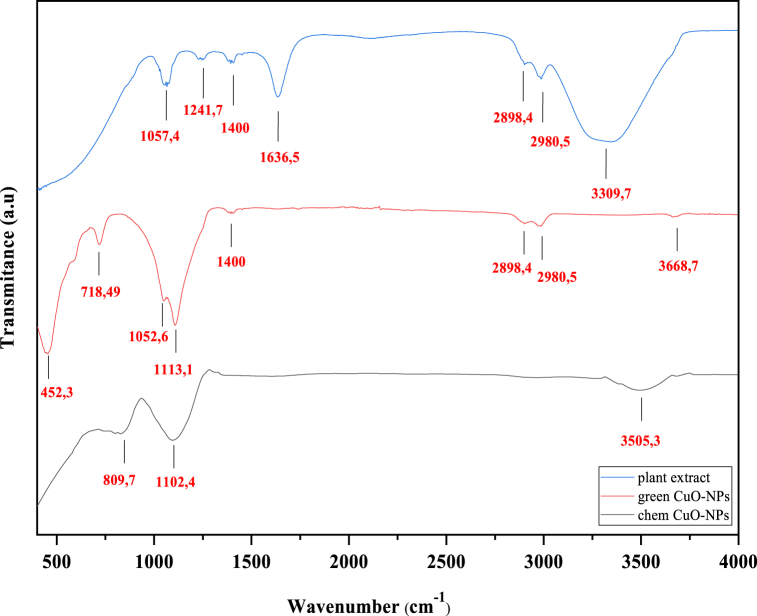
FTIR spectra of *E. alata* extract plant, green synthesized CuO-NPs and chemical CuO-NPs. (For interpretation of the references to color in this figure legend, the reader is referred to the Web version of this article.)

The FTIR technique was realized in the range of 400–4000 cm^−1^ in order to understand the role played by the extract of the plant *E. alata* as a reducing and capping agent of new functional groups and biomolecules attached to the surface of CuO-NPs. The FTIR spectrum showed the different absorption peaks of chemical CuO-NPs and biosynthesized CuO-NPs (Fig. 3). The chem CuO-NPs showed absorption peaks of 3505.3, 1102.4, and 809.7 cm^−1^. The band around 3505.3 cm^−1^ corresponds to bending vibration-free O–H [17,20]. A strong peak at 1102.4 cm-1 can be attributed to the vibrations of CuO, confirming the formation of CuO nanoparticles [58]. The peak at 809.7 cm^−1^ is assigned to the M-*O*-M stretching mode of vibration (M = Cu) [17,59]. For biosynthesized CuO-NPs, the absorption peaks observed at 452.3 cm^−1^ are produced by asymmetric stretching, which is related to a deformation vibration of Cu–O in the (−202) direction that indicates the presence of monoclinic CuO [6,60]. The absorption peaks noted at 718.49 and 1052.6 cm^−1^ are due to the M − O stretching vibrations of CuO (M = Cu) [61]. The strong peak at 1113.1 cm^−1^ is attributed to CuO vibrations [62], whereas that at 1400 cm^−1^ corresponds to the OH phenolic bending [4]. The bands around 2898.4 and 2980.5 cm^−1^ are related to C–H stretching [4]. It is attributed to the presence of phenolic and flavonoid groups attached to the surface of the nanoparticles after the synthesis process [63]. The small peak at 3668.7 cm^−1^ is due to hydroxyl groups [64]. From the FTIR analysis, it was supposed that the phenol and flavonoides present in the plant extract were responsible for the reduction of the metal salt to metal oxide NPs and acted as stabilizing and capping agents for the CuO NPs. Thus, various phytochemicals in the plant extract, such as phenols and flavonoides, are implicated in the synthesis of CuO NPs. Indeed, these FTIR peaks are consistent with the crystalline biosynthesized CuO-NPs pure phase characterized by XRD.

### 3.3 Optical study

**Fig. 4:**
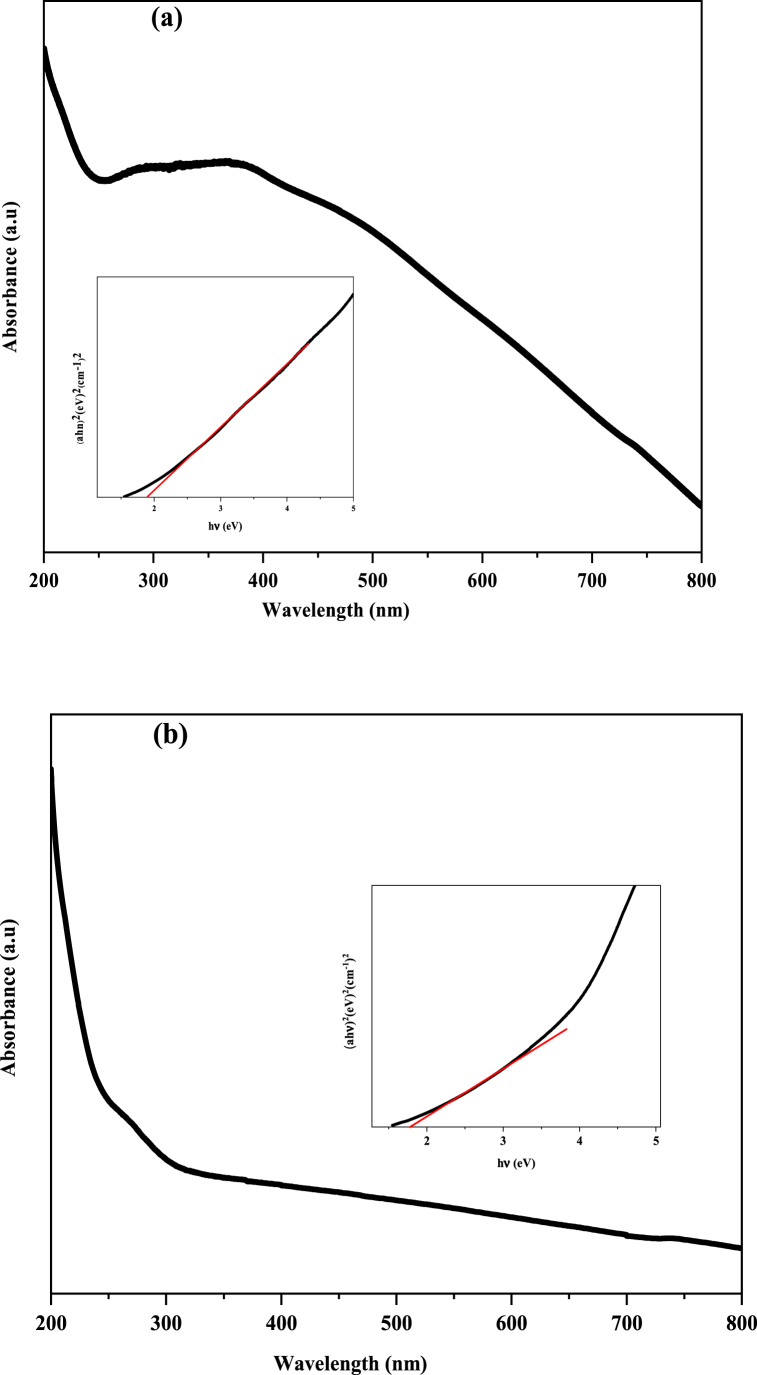
UV–Visible spectra and optical bandgap energy of Chem CuO-NPs (a) and Biosynthesized CuO-NPs (b).

The optical absorption properties of synthesized copper oxide nanoparticles (CuO) were examined by UV-VIS spectroscopy in the wavelength range of 200–800 nm. The spectra of chem CuO-NPs and biosynthesized CuO-NPs are presented in Fig. 4(a,b). The spectra of the chemical CuO-NPs showed a wide absorption band in the region of 280–410 nm. The spectra of chemical CuO-NPs displayed a broad absorption band, which can be due to the presence of an impurity of cuprous oxide nanoparticles (Cu2O) [65,66]. However, the biosynthesized nanoparticles using *E. alata* extract exhibted an absorption peak at about 272 nm, confirming the good formation and pure phase of CuO-NPs [4,62]. The presence of components such as flavonoids and polyphenols in the plant extract is accountable for the conversion of the copper salt to CuO-NPs, and confirmed the FTIR spectra analysis [28,64]. The band gap of synthesized CuO-NPs was estimated by the tauc [67] model Eq. (5), which extrapolates as a function of hν, as shown in Fig. 4a,b.

(5) $$\alpha h\upsilon =k{\left(h\upsilon -E_{g}\right)}^{1/2}$$

where hν is the light energy, αis the absorption coefficient, E_g_ is the direct band gap, k is a constant, and n is the transition index. The band gap energies were calculated to be 1.92 and 1.77 eV for chemical CuO-NPs and biosynthesized CuO-NPs, respectively. This indicates that the gap energy (E_g_) of biosynthesized CuO-NPs decreased compared to chemical CuO-NPs.

### 3.4 FE-SEM

**Fig. 5:**
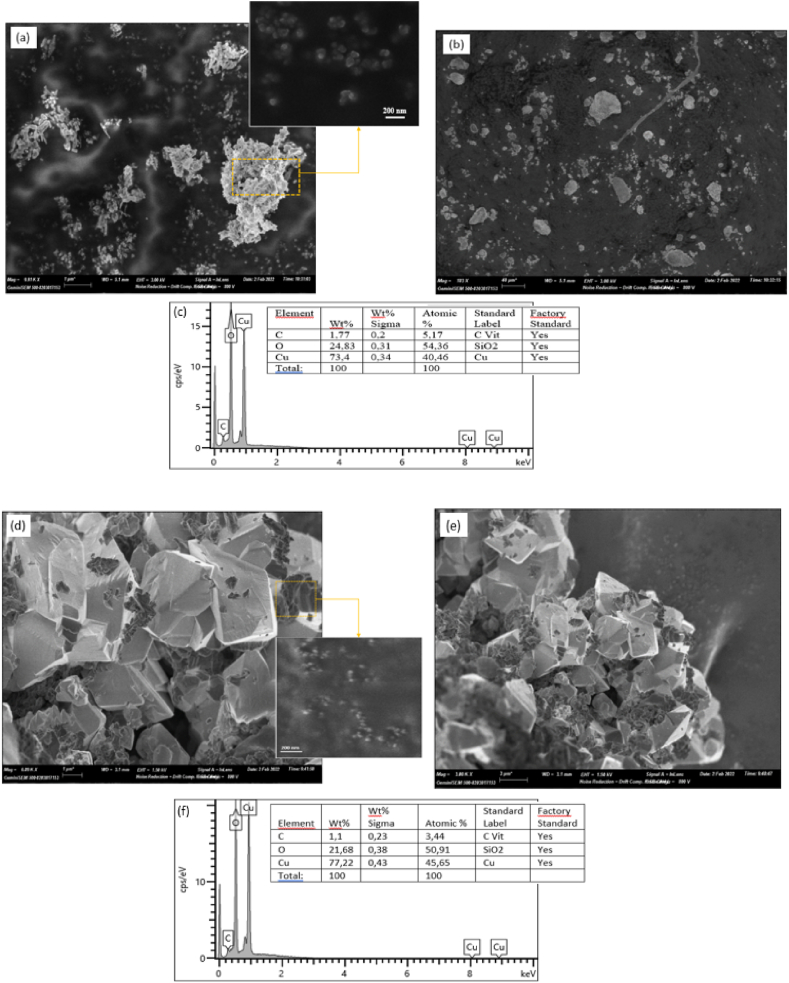
FE-SEM images and EDX Spectra of synthesized CuO-NPs at different magnifications (a,b et c): chem CuO-NPs and (d,e et f): Green CuO-NPs. (For interpretation of the references to color in this figure legend, the reader is referred to the Web version of this article.)

FE-SEM and EDX images of chemically synthesized CuO nanoparticles and green chemistry synthesized CuO nanoparticles are shown in Fig. 5. FE-SEM images (Fig. 5 (a,b)) of the chemical CuO-NPs showed the appearance of a few mainly spherically-shaped crystals, which can be explained by slight agglomeration. The green CuO NPs revealed that the powders are composed of octahedral crystal clusters and some spherical nanoparticles. This agglomeration is due to the oxidation of metal nanoparticles and the overlapping of small crystals (Fig. 5 (d,e)). This can be caused by the coating of various surface active groups from the prepared extracts [20,63]. On the other hand, the elements present in the prepared CuO NPs were confirmed by energy dispersive X-ray (EDX) and the observed peaks are shown in Fig. 5 (c,f). It is observed that the synthesized CuO NPs are principally constituted by Cu, O and C without any trace of other elements, which confirms the purity of the copper oxide without other impurities. No other components were present, even from the extract. In both samples, the EDX spectra indicated a high signal peak at 1 keV corresponding to copper atoms [19,68].

### 3.5 Photocatalytic activity performance of CuO NPs

**Fig. 6:**
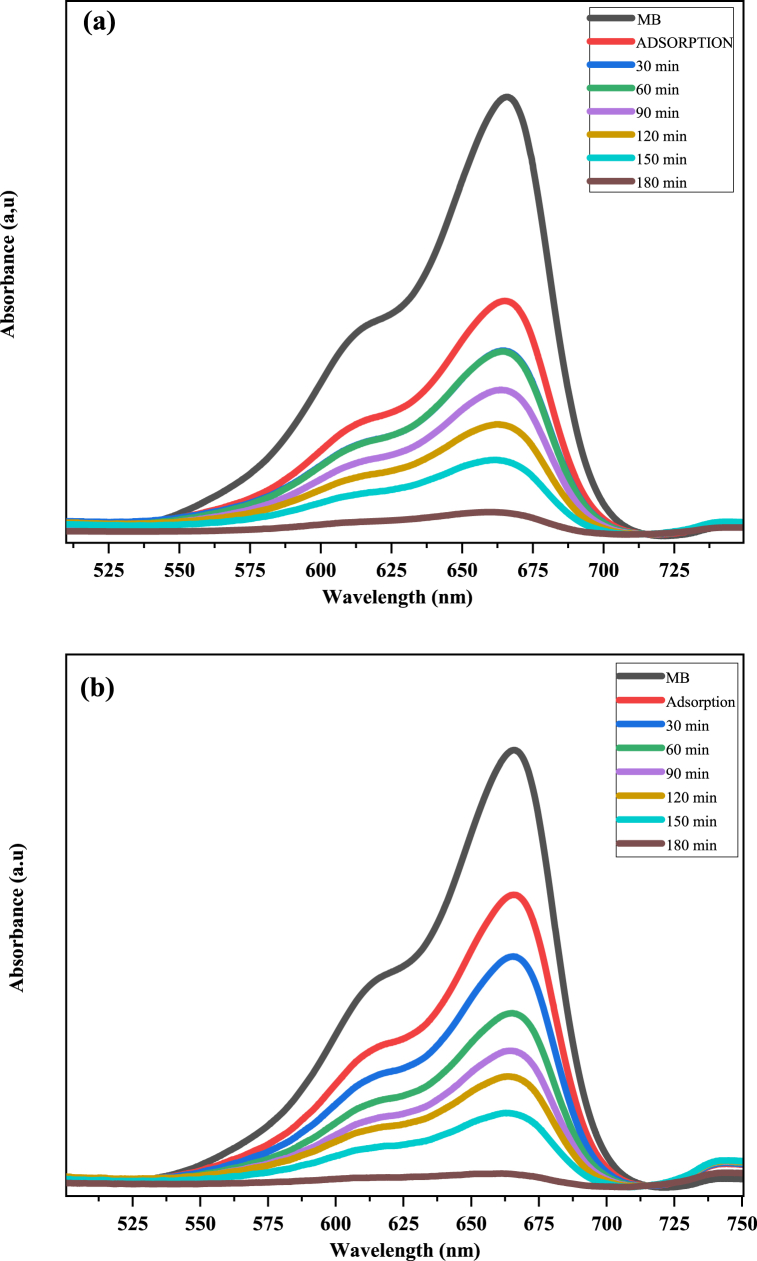
UV–Vis absorption spectra of photocatalytic degradation of methylene blue with respect to irradiation time (a: chem CuO-NPs and b: Green CuO-NPs). (For interpretation of the references to color in this figure legend, the reader is referred to the Web version of this article.)

**Fig. 7:**
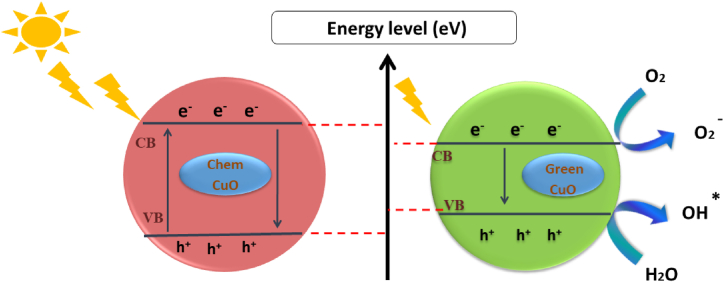
Mechanism for degradation of MB dye by chem CuO-NPs and biosynthesized CuO-NPs.

The photocatalytic activity of the chemical and biosynthesized CuO NPs was investigated for the degradation of aqueous solutions of methylene blue under sunlight irradiation. The degradation of MB dye is shown in Fig. 6 (a,b). The UV-VIS spectrophotometer is noted from 500 to 750 nm for various time ranges (0–180) min. The absorption peak was noticed at a wavelength of 666 nm of MB dye, which is detected by the change in color of the solution from blue to uncolored due to electron transfer (Fig. S2). The extent of degradation was measured at absorbance at 666 nm and indicated that 80.2 and 93.4% of MB dye degraded in precisely 180 min, respectively, by the chem CuO-NPs and biosynthesized CuO-NPs. This result reveals the high photocatalytic activity of the biosynthesized CuO-NPs for the degradation of MB dye in comparison with that of the chem CuO-NPs. This might be caused by a decline in the crystalline quality and perhaps even a reduction in the average crystallite size of the chemically produced particles (Fig. 2), which would lead to a decrease in the photocatalytic degradation of MB [50]. The enhanced rate of reduction by the biosynthesized CuO-NPs is attributed to the transitional redox potential between the donor and the acceptor over the electron moving process [69,70]. On the one hand, the catalytic reduction was due to the large surface area, which improved the interaction between reactants and the catalyst surface and facilitated degradation [30,71]. On the other hand, biosynthesized CuO-NPs are considered an effective redox catalyst due to the electron relay effect in the MB reduction reaction. The photodegradation mechanism for the degradation of MB dye using chem CuO-NPs and bio CuO-NPs is summarized in Fig. 7. After the addition of CuO-NPs to the dye solution, the dye molecules adsorbed on the NPs surface. The CuO NPs interacted with the irradiation of the sunlight and generated electrons and holes pursued by a photochemical reaction. Pairs were created (e^−^/h^+^) and electrons from the valence band were excited to the conduction band where holes were located at the VB. Then, the electrons reacted with the surface of the nanoparticles and the photo-induced electrons were easily captured by electronic acceptors, such as adsorbed O_2_, to create a super oxide anion radical. The positive hole in the NPs reacted with $H_{2}O$ to produce hydrogen and the free hydroxyl radical, which is the radical most responsible for the mineralization of adsorbed or free MB molecules on the surface of CuO-NPs. The first stage of MB degradation involves the attack of OH on the C–S+=C functional group. The central aromatic ring, which contains the heteroatoms S and N, is opened to preserve the double bond conjugation that was lost during the transition from C–S+=C to C–S (=O)–C. CH and NH bonds formed because of hole-induced H+. The main cause of dye degradation is the splitting of a complex molecule into smaller and highly oxidized intermediate molecules. Additionally, the MB dye may be attacked directly by the VB holes. The dye can be directly oxidized by holes due to their high oxidation potential, which leads to reactive intermediates that can be degraded [72,73]. Furthermore, the free MB molecules absorb solar radiation, which allows their excitation and functions as a catalyst for the degradation of MB molecules [50,[74], [75], [76]]. The photocatalytic degradation mechanism of CuO-NPs based methylene blue dye irradiated by sunlight was presented in a previous work [77] and is described below:

$$CuO+h\upsilon \to CuO\left(e^{-}+h^{+}\right)$$

$$CuO+h\upsilon \to CuO\left(e^{-}\right)+CuO\left(h^{+}\right)$$

$$CuO\left(e^{-}\right)+O_{2}\to O_{2}^{-}$$

$$CuO\left(h^{+}\right)+H_{2}O\to OH^{-}$$

$$O_{2}^{-}orOH^{-}+Dye\to Degraded\;product$$

The surface of CuO-NPs was enriched by the phenol and flavonoid groups of *E. alata* plant extract, which can enhance the free oxygen to superoxide radicals, thereby improving the photocatalytic efficiency [78]. The performance of the MB degradation kinetic procedure exanimated by the first-order kinetic model, which is one of the favorable models to demonstrate the kinetic behavior of a photocatalyst, can be expressed as [79].

$$\mathrm{Ln}\left(\frac{A_{0}}{A_{t}}\right)=\mathrm{Ln}\left(\frac{C_{0}}{C_{t}}\right)=-kt$$

**Fig. 8:**
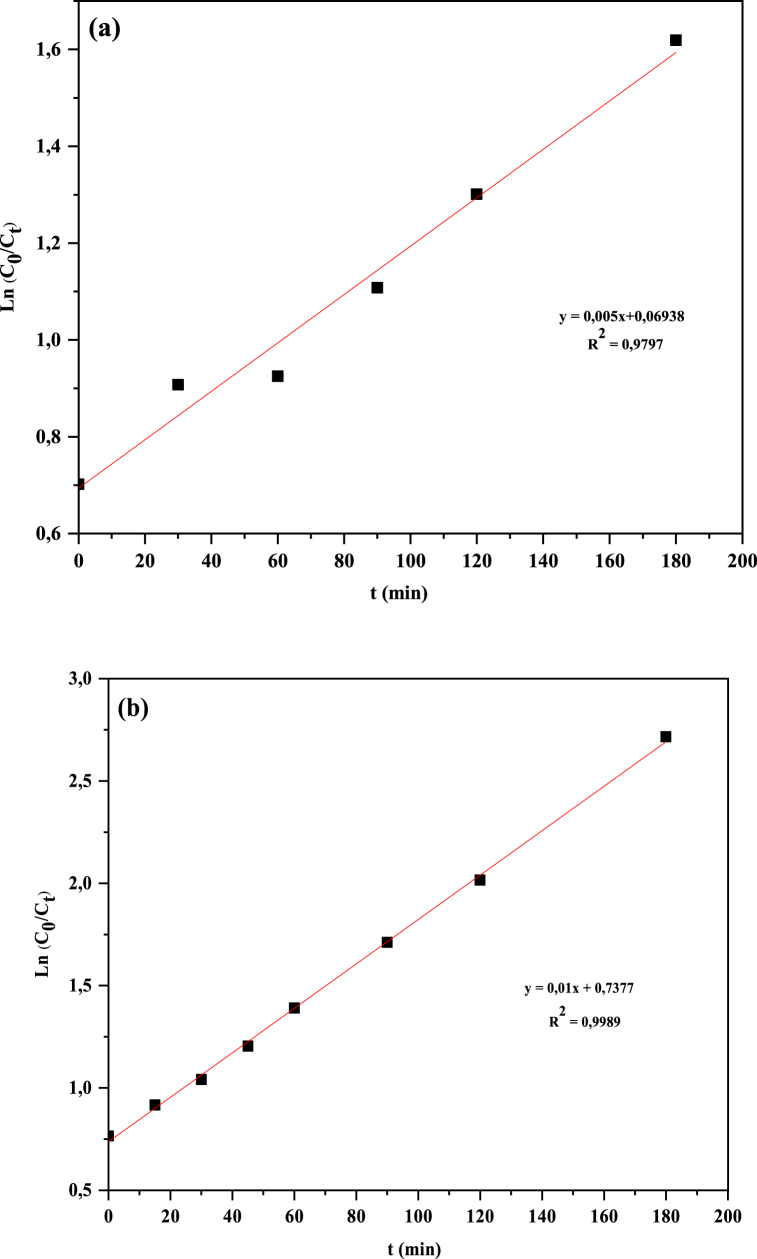
Plot of ln (C0/Ct) versus time for the degradation of MB by chem CuO-NPs (a) and biosynthesized CuO-NPs (b).

Where, A_0_ and A_t_ are the ratio of the absorbance of MB at time = 0 and t respectively, C_0_ and C_t_ are the initial concentration and the concentration after any time t of aqueous MB respectively, k is the obvious first-order rate constant for the reaction (min ^−1^), and t is the response time in minutes. The kinetic of photodegradation of MB by chem CuO-NPs and biosynthesized CuO-NPs were investigated and the results are presented in Fig. 8 (a,b). Therefore, the rate constant (k) for MB dye degradation by synthesized CuO-NPs was calculated by the pseudo first order equation. The evolution of versus time showed good linear correlation with the values coefficient (R^2^) determined to be 0.9797 and 0.9989 for chem CuO-NPs and biosynthesized CuO-NPs. Then, the slope of the fitting line determined the rate constant values 0.005 min ^−1^ for chem CuO-NPs and 0.01 min ^−1^ for biosynthesized CuO-NPs. The biosynthesized CuO-NPs sample showed higher *k* value than chemical CuO-NPs. Under the excitation of sunlight, the phenol and flavonoid groups present in the extract promoted the transfer of electrons to the conduction band of CuO, which enhances the conversion of oxygen to superoxide radicals and thus accelerates the photodegradation of biosynthesized CuO-NPs catalysts [78].

**Fig. 9:**
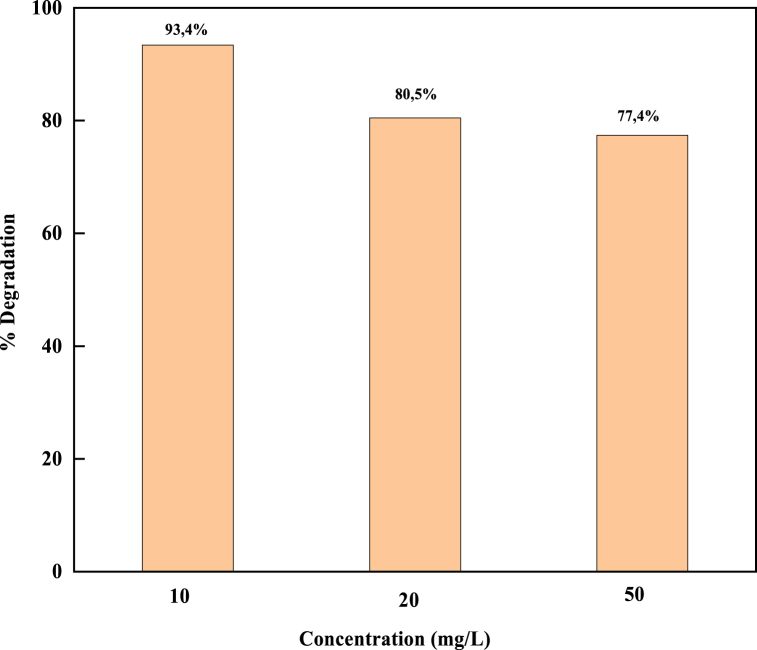
The effect of the initial MB concentration on the biosynthesized CuO-NPs.

The initial dye concentrations of 10, 20, and 50 mg/L were changed to observe the impact of the initial MB concentration on the biosynthesized CuO-NPs catalyst (Fig. 9). It was discovered that the relationship between the photodegradation efficiency of MB and its concentration is inverse. The photodegradation efficiency of NPs decreased with the rise of dye concentrations from 10 to 50 mg/L. The generation of hydroxyl radicals, a key species in the degradation process, is necessary for photodegradation to occur efficiently. Because of the static concentration of the catalyst, only fewer active sites for OH^−^ adsorption exist, implying a decrease in OH^−^ generation. Additionally, with the rise of MB concentration under illumination and constant light intensity, fewer photons can reach the catalyst surface due to shorter photon paths entering the solution. As a result, fewer holes and hydroxyl radicals were produced, which can attack methylene blue. Consequently, the relative amount of OH^−^ attached to the compound decreases, reducing the effectiveness of photodegradation [[80], [81], [82]]. The ideal initial dye concentration is therefore 10 mg/L with a degradation rate of 93.4%.

**Fig. 10:**
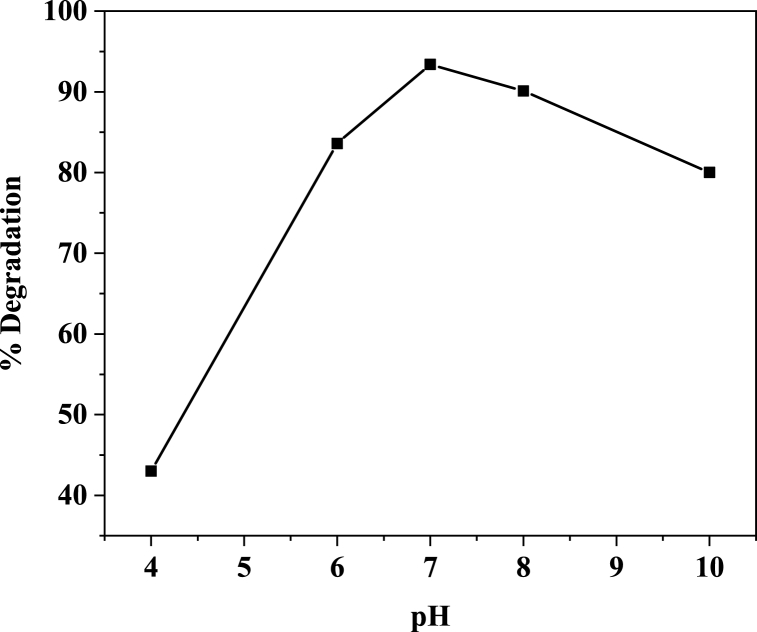
pH effect on MB dye degradation using biosynthesized CuO-NPs.

**Fig. 11:**
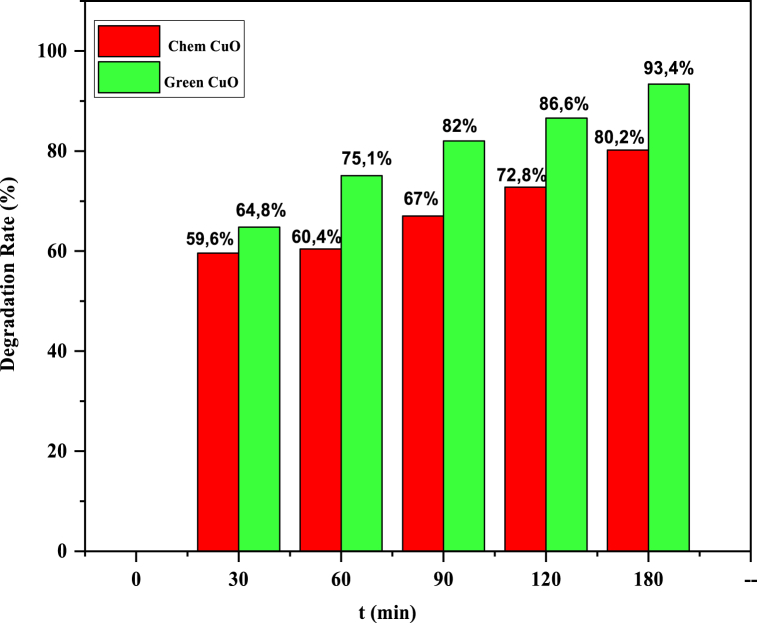
% Degradation rate of MB dye compared to the chem CuO-NPs and biosynthesized CuO-NPs.

Fig. 10 shows the effect of solution pH on the degradation percentage of MB in the aqueous solution. Based on these results,a pH equal to7 is the optimal pH for the degradation of MB using CuONPs made using a green method. Additionally, it has been noted that the pHpZc of the catalysts in literature is around 7, and at this pH, the environment neutralizes the charge on the surfaces of the catalysts [83,84]. At pH < pHpzc, the catalyst surface charge is positive, at acidic pHs, cationic MB molecules should be repelled. Therefore, little degradation is anticipated. At pH values between 4 and 6, it was discovered that MB degradation increased. At a pH greater than pKa (3.8), the majority of the MB is present as neutral. In this situation, the amount of MB degradation increased by the attractive force between the MB molecules and the positively charged catalyst surface. The negatively charged surface of the catalyst repels MB molecules at pHs greater than pHpzc, which reduces the amount of degradation [85]. The histogram (Fig. 11) showed that the degradation in the performance of MB decreases with increasing dye concentration. Depending on the results obtained (Table 2), the *E. alata* extract using biosynthesized CuO-NPs indicated better photocatalytic activity of MB dye than chem CuO-NPs and the other plant extract.

**Table 2:** Comparison of degradation efficiencies of MB dye using NPs prepared from different plant extract.

Catalyst	Biological entity	Dye	%Degradation	Time	Reference
CuO-NPs	*Lemongrass leaf*	MB	80	300 min	[79]
CuO-NPs	*Solanum lycopersicum leaf*	MB	97	300 min	[86]
CuO-NPs	*Madhuca longifolia leaf*	MB	77	120 min	[87]
ZnO-NPs ZnS-NPs ZnO- NPs CuO-NS	*Azadirachta indica* Chemically E. Japonica leaves aqueous extract of *Rhazya stricta*	MB MB MB MB	82 49 72 82.7	120 min 180 min 160 min 140 min	[88] [89] [90] [77]
CuO-NPs	Chemically	MB	80.2	180 min	Present work
CuO-NPs	*Ephedra Alata leaf*	MB	93.4	180 min	Present work

### 3.6 Antibacterial activity

The in vitro antibacterial activity was realized using the disk diffusion technique against Gram-negative bacteria: *S. aureus* and *B. subtilis* for chemically synthesized CuO nanoparticles and CuO nanoparticles synthesized with *E. alata* plant extract. In this procedure, CuO-NPs (test samples), antibiotic discs (as positive controls), and distilled water (as negative controls) were tested against the bacterial strains. However, they concluded that the green synthesized copper oxide nanoparticles exhibited higher antibacterial activity with average inhibition zones of 20.4 and 16 mm compared to chemically synthesized copper oxide nanoparticles, which had inhibition zones of 16 and 11.6 mm for *S. aureus* and *B. subtilis*, respectively (Table .3). Interestingly, the antimicrobial efficacy of vigorously synthesized green CuO-NPs versus chemically synthesized CuO-NPs is due to their morphologies and biocompatible nature to treat a large range of human pathogenic bacteria. The obtained CuO NPs' antibacterial impact may be explained by taking into account their small size and extraordinarily high surface area/volume ratio, which creates superior circumstances for the interaction with bacterial cells. Additionally, the Cu2+ ions might be readily released from such CuO NPs, allowing them to interact with intercellular biomolecules like DNA and proteins and enter bacterial cell membranes more effectively. These processes can reduce the viability of the cells and lead to cell death [25,40,[91], [92], [93], [94]]. The results are in agreement with other research reporting a significant impact of synthesized green copper oxide nanoparticles against bacterial strains [18,20,95].

**Table 3:** The inhibition zone of the chem CuO-NPs and biosynthesized CuO-NPs against bacterial and fungi strains.

Microorganisms	Zone of growth inhibition (mm)					
	Green CuO-NPs			Chemical CuO NPs		
**Bacteria**rowhead						
*S. aureus* ATCC 6538	20.4		16			
B. subtilis ATCC 6633	16		11.6			
**Fungi**rowhead						
*C. albicans* ATCC 10231	16.2	–				
*S. cerevisiae* ATCC 9763	18.4	–				

### 3.7 Antifungal activity

The antifungal activities of chemical and green CuO NPs against *C. albicans* and *S. cerevisiae* were determined using the disc diffusion method. Green CuO NPs had stronger antifungal activity than that of chemical CuO NPs against *C. albicans* and *S. cerevisiae*, where the inhibition zones of green CuO NPs were 16.2 and 18.4 mm, respectively (Table .3). This result is really important to show the effect of the biosynthesis method on enhancing the potential of antifungal activity of metal oxide nanoparticles [22,95,96].

## 4 Conclusion

We report a simple chemically and biosynthesized route for the synthesis of nanoparticle copper oxide using *E. alata* extract as a reducing and stabilizing agent. XRD pattern of synthesized CuO-NPs exhibted the monoclinic structure with a uniform size distribution of 10–16 nm. The FTIR spectra indicated the presence of the molecules of phenol and flavoinoides in the surface of CuO-NPs. UV–Visible spectra showed the band gap energy of chemically and boisynthetesized CuO-NPs was found to vary from 1.77 to 1.92 eV for chemically and green synthesized CuO respectively. The morphology of CuO-NPs reveals the spherical shaped crystals of the chem CuO-NPs, while the green CuO NPs reveal octahedral crystal clusters with different diameters. The photocatalysis, antibacterial and antifungal were studied for CuO chemically and boisynthetesized. It confirmed that the green CuO-NPs possessed an improved photocatalytic degradation of MB dye under direct sunlight, antibacterial activity against negative gram *as S. aureus* and *B. subtilis*, and strong antifungal activity against *C. albicans* and *S. cerevisiae*. The biosynthesized route is important than chemically because of it offered a high purty, high active site, simplicity, low cost, non-toxicity, eco-friendly, and low energy consumption. It is concluded that the biogenic synthesized of CuO-NPs with *E. alata* extract is best condidats for remediation of polluted water and biomedical applications.

## Author contribution statement

Afrah Atri: Performed the experiments; Analyzed and interpreted the data; Wrote the paper.

Mosaab Echabaane: Conceived and designed the experiments;Analyzed and interpreted the data.

Amel Bouzidi: Analyzed and interpreted the data; Contributed reagents, materials, analysis tools or data.

Imen Harabi: Contributed reagents, materials, analysis tools or data.

Bernabe Mari Soucase: Contributed reagents, materials, analysis tools or data.

Rafik Ben Chaâbane: Conceived and designed the experiments.

## Funding statement

This work was supported by Tunisian's Ministry of high education and scientiﬁc research.

## Data availability statement

The data that has been used is confidential.

## Declaration of interest’s statement

The authors declare no conflict of interest.
