# Supplementary material for: Green synthesis of copper oxide nanoparticles using Ephedra Alata plant extract and a study of their antifungal, antibacterial activity and photocatalytic performance under sunlight
Source: Heliyon. 2023 Feb 4;9(2):e13484. doi: 10.1016/j.heliyon.2023.e13484 (PMC9929317; doi:10.1016/j.heliyon.2023.e13484)
Supplement: Multimedia component 1 [file mmc1.docx]

**Support information**


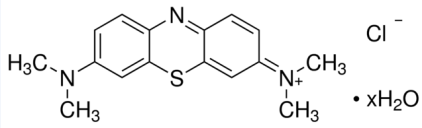


**Fig. S1.** The chemical structure of MB dye.

**Table S1**. Characteristics of MB dye

| Dye Molecular formula |  | Molecular weight (g/mol) λ_max_(nm) |  |
| --- | --- | --- | --- |
| Methylene blue C_16_H_18_ClN_3_^.^ xH_2_O |  | 319.85 666 |  |


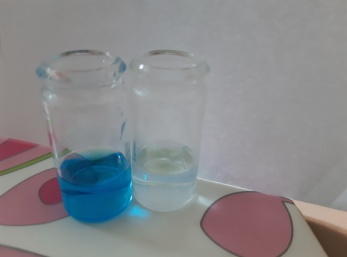


**Fig.S2.** Gradual change in dye color from blue to colorless
